# Supplementary material for: Genome-wide identification and analysis of recurring patterns of epigenetic variation across individuals
Source: Commun Biol. 2025 Jun 7;8:888. doi: 10.1038/s42003-025-08179-5 (PMC12145423; doi:10.1038/s42003-025-08179-5)
Supplement: Supplementary file 1 — Supplementary Figs. 1–28 and Supplementary Table 1 [file 42003_2025_8179_MOESM1_ESM.pdf]

Genome-wide identification and analysis of recurring patterns of epigenetic variation across individuals

Jennifer Zou[1,7], Emily Maciejewski[1,2,7], and Jason Ernst[1,2,3,4,5,6,*]

[1] *Computer Science Department, University of California Los Angeles, Los Angeles, CA, 90095, USA*

[2] *Biological Chemistry Department, University of California Los Angeles, Los Angeles, CA, 90095, USA*

[3] *Department of Computational Medicine, University of California Los Angeles, Los Angeles, CA, 90095, USA*

[4] *Molecular Biology Institute, University of California Los Angeles, Los Angeles, CA, 90095, USA*

[5] *Eli and Edythe Broad Center of Regenerative Medicine and Stem Cell Research at University of California Los Angeles, Los Angeles, CA, 90095, USA*

[6] *Jonsson Comprehensive Cancer Center, University of California Los Angeles, Los Angeles, CA, 90095, USA*

[7] *These authors contributed equally*

[*] *Email: jason.ernst@ucla.edu*

Content

Supplementary Figures 1-28

Supplementary Table 1

Supplementary information

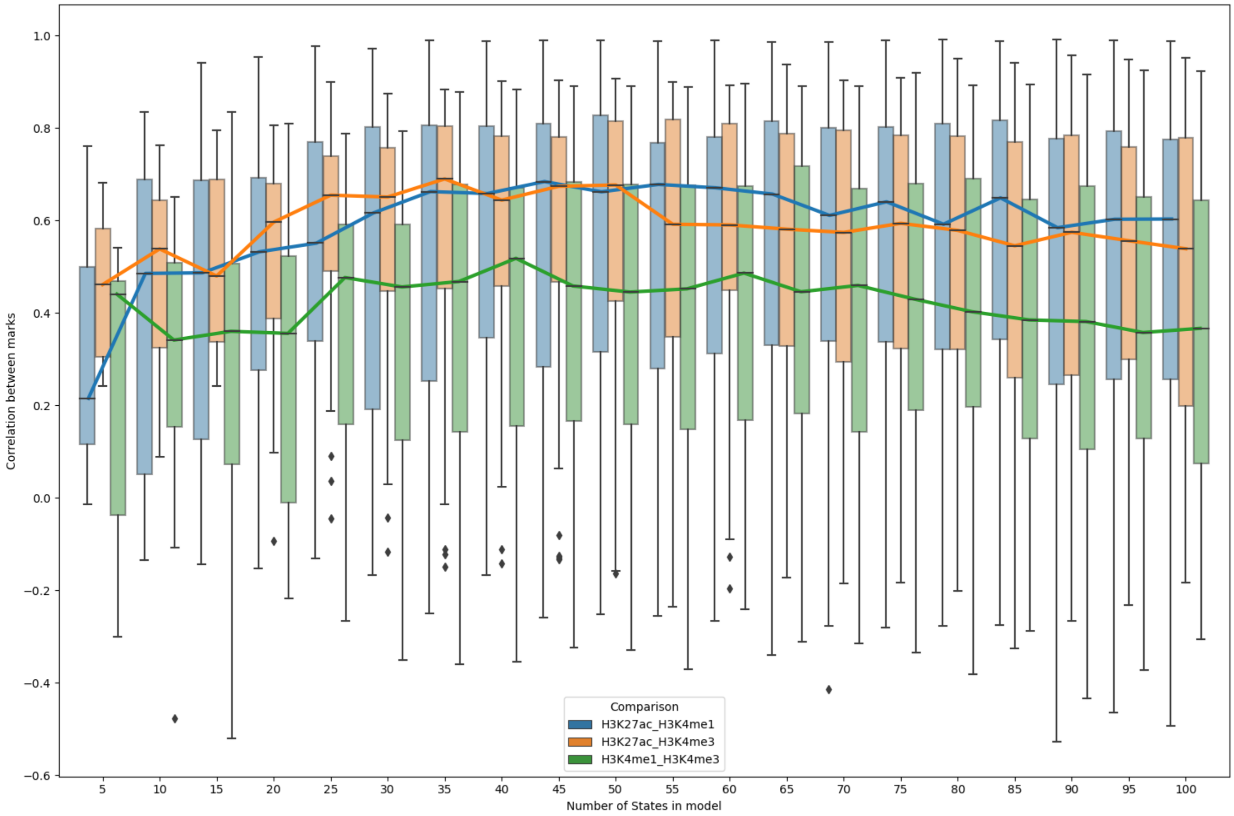

Supplementary Fig. 1: **Pairwise correlation of marks in the LCL data set.** For each pair of histone modifications (H3K27ac, H3K4me1, H3K4me3), we computed the median Spearman correlation of the emission parameters across individuals (n=75) within each state. Each colored line corresponds to a pair of marks. The median pairwise correlation (y-axis) is shown as a function of the number of states used to train each model (x-axis). The boxplots represent the distribution of correlations. The center line of the boxplot corresponds to the median emission across samples. The box limits correspond to the upper and lower quartiles.

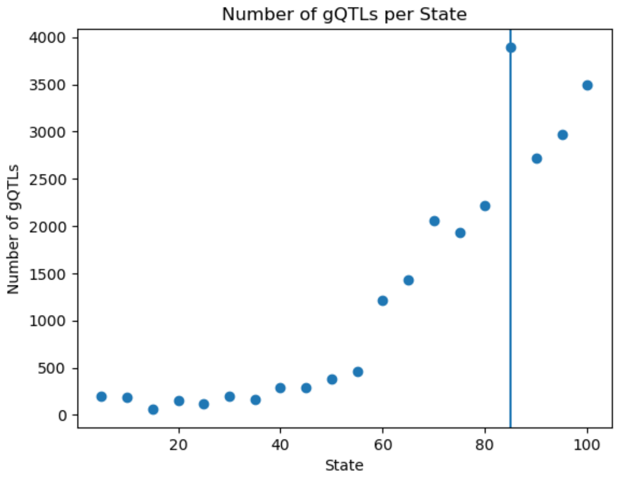

Supplementary Fig. 2: **LCL model gQTLs.** The total number of significant gQTLs (FDR<5%) in the LCL model (y-axis) is shown as a function of the number of states (x-axis). The number of gQTLs is maximized using an 85-state model denoted with the vertical line.

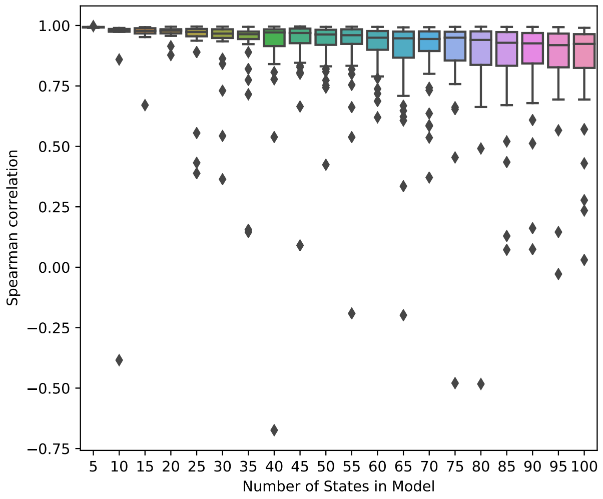

Supplementary Fig. 3: **Pairwise state correlations in the LCL data set.** For each number of states (x-axis), two models were trained on different subsets of data (Methods). Each dot corresponds to the Spearman correlation (y-axis) of the emission parameters of a state in one model with a paired state in the other model. States between the two models were paired using a greedy algorithm (Methods). The center line of the boxplot corresponds to the median pairwise correlation across states. The box limits correspond to the upper and lower quartiles.

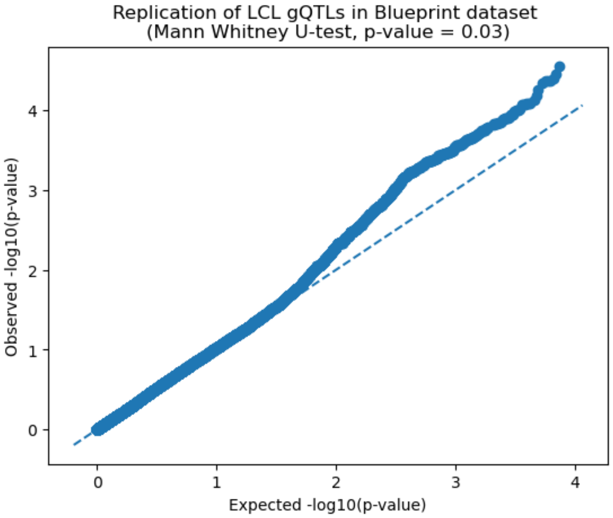

Supplementary Fig. 4: **LCL gQTL replication analysis.** Results from a replication analysis on 739 of the 2945 gQTLs identified in the LCL data that overlap in the BLUEPRINT dataset. Expected $-log10(p\text{-}values)$ (x-axis) were computed using theoretical values from a uniform distribution. Observed $-log10(p\text{-}values)$ (y-axis) were computed in the replication analysis. The dashed line corresponds to the null, where p-values from the replication experiment have the same quantiles as those from a uniform distribution. The distribution of the replication p-values was significantly lower than we would expect by chance (Mann Whitney U-test $p = 0.03$).

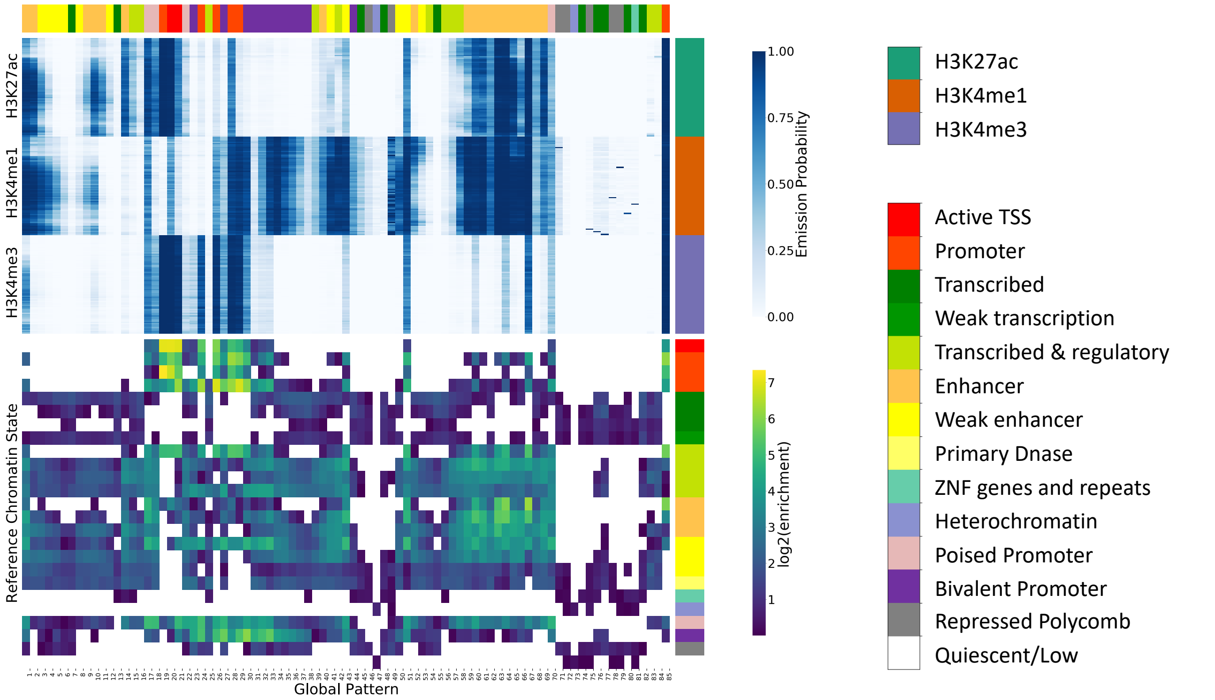

Supplementary Fig. 5: **Fold enrichment of chromatin states for LCL 85-state model.** The top heatmap shows the emission parameters learned for the 85-state model using the LCL dataset. The global patterns are on the x-axis, and the datasets are on the y-axis. The datasets are grouped by histone modification, and the individuals have the same ordering within each mark. The bottom heatmap shows the log2 fold enrichment of a previous reference chromatin state annotation in LCL for one individual based on imputed data for 12-marks, where states with the same annotation color (left) represent different sub-states of the same category[1]. Significant enrichments are indicated with color (Binomial Test (two-sided), FDR<5%). The global patterns are shown on the x-axis ("Global Pattern"), and the LCL reference chromatin states on the y-axis ("Reference Chromatin State"). Each global pattern is annotated with the highest enriched chromatin state from the reference individual (top).

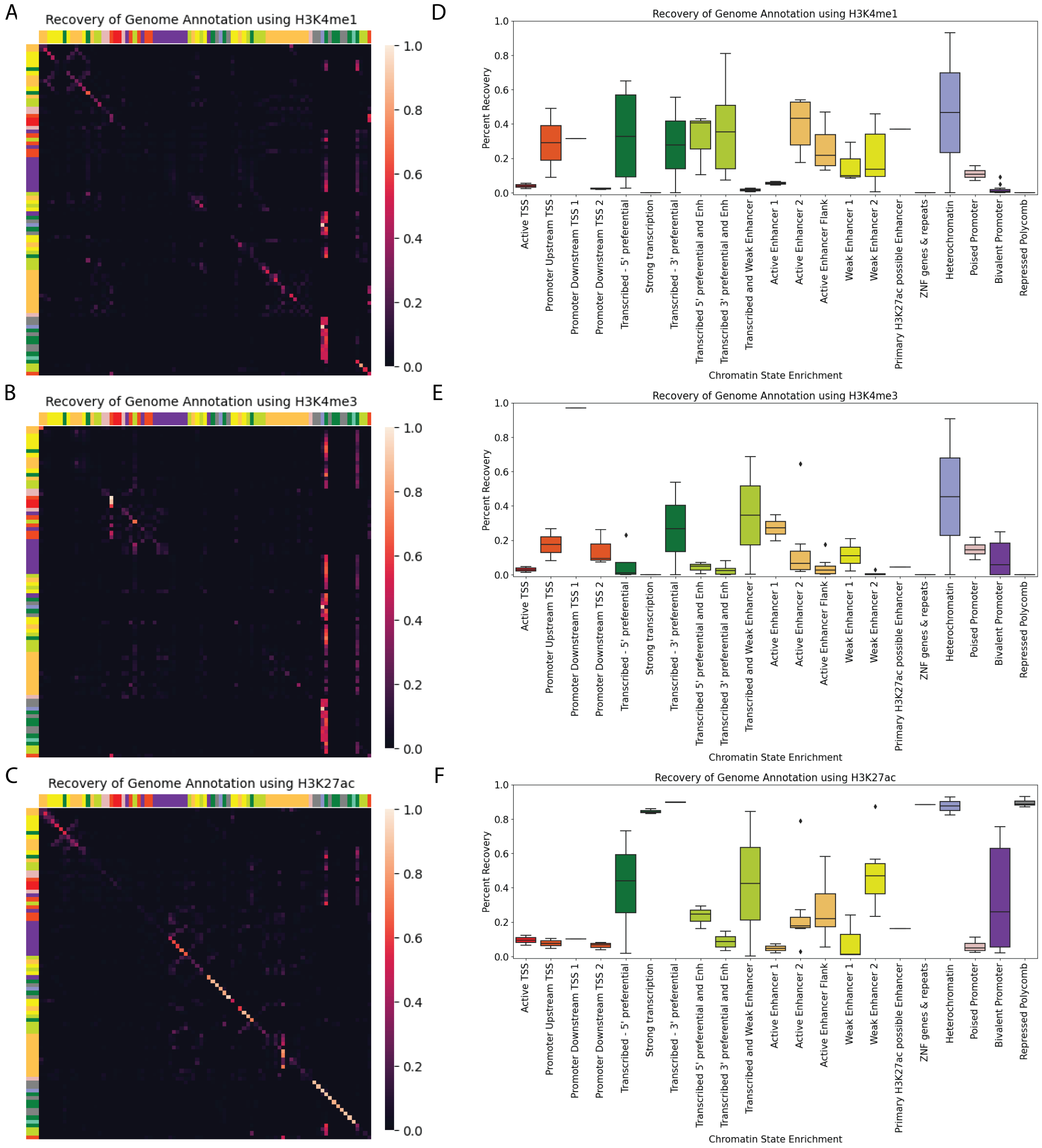

Supplementary Fig. 6: **Recovery of global pattern genome annotation using one histone modification. A-C)** Heatmaps displaying the confusion matrices when using only data from a single mark: **A)** H3K4me1, **B)** H3K4me3, and **C)** H3K27ac to annotate global patterns based on the LCL model learned using all three marks. Each row corresponds to a state in the original model trained on all three histone modifications. Each column represents the percentage of the genome annotation assigned to each state when using the indicated mark for decoding. The diagonal of the confusion matrix represents the percentage of the original annotation that was recovered based on the single mark. The color bar labels each state by the chromatin state annotation similar to Figure 2a. **D-F)** Boxplots showing the diagonal values of the confusion matrices when using only data from a single mark on **D)** H3K4me1, **E)** H3K4me3, and **F)** H3K27ac to annotate global patterns with the LCL model learned using all three marks. Each box corresponds to the global patterns enriched for a chromatin state annotation. The boxes use the same color scheme as the color bars in A-C and Figure 2a.

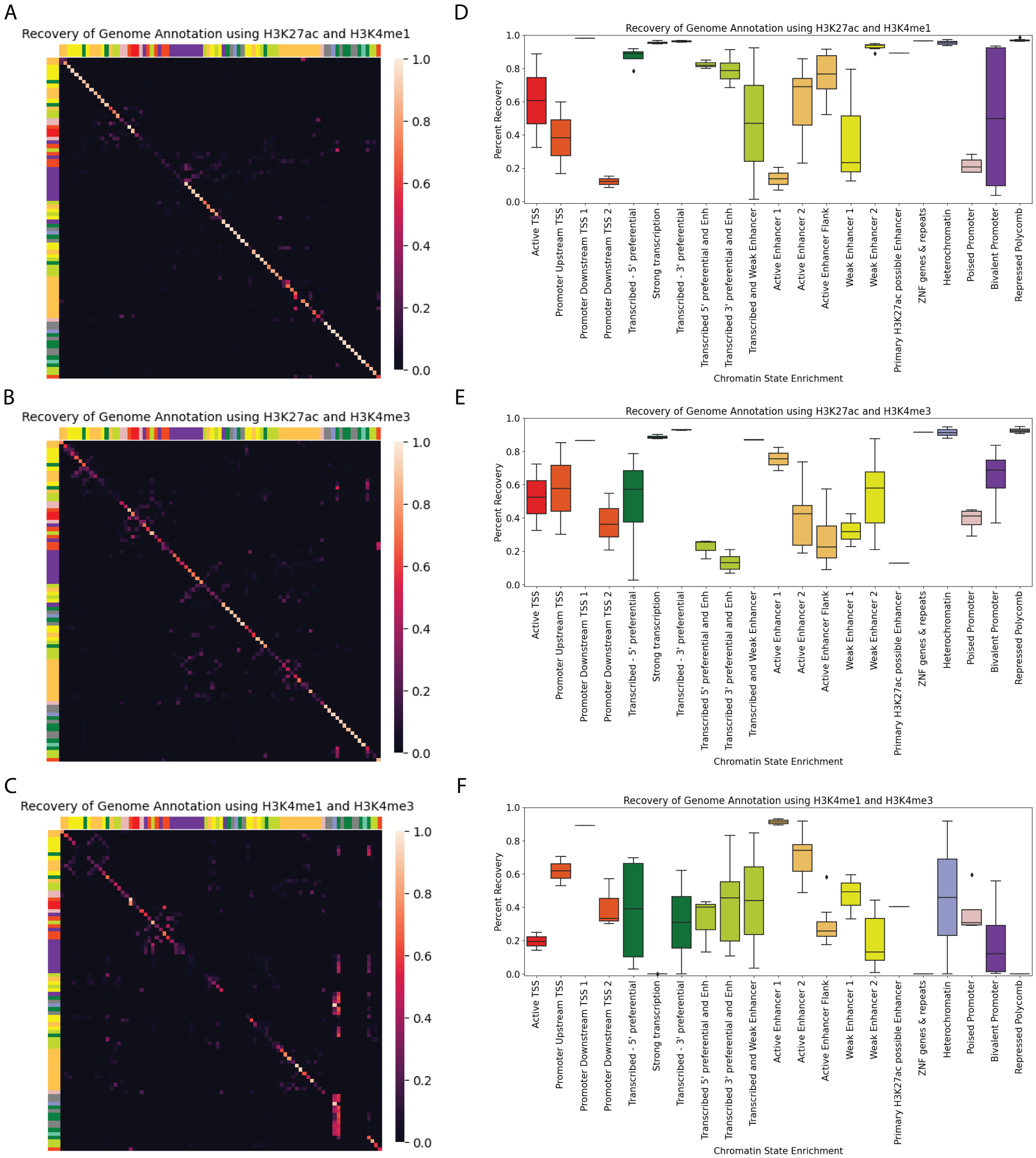

Supplementary Fig. 7: **Recovery of global pattern genome annotation using two histone modifications. A-C)** Heatmaps displaying the confusion matrices when using only data from pairs of marks: **A)** H3K27ac and H3K4me1, **B)** H3K27ac and H3K4me3, and **C)** H3K4me1 and H3K4me3 to annotate global patterns based on the model learned using all three marks. Each row corresponds to a state in the original model trained on all three histone modifications. Each column represents the percentage of the genome annotation assigned to each state when using the indicated mark for decoding. The diagonal of the confusion matrix represents the percentage of the original annotation that was recovered based on the pair of marks. The color bar labels each state by the chromatin state annotation similar to Figure 2a. **D-F)** Boxplots showing the diagonal values of the confusion matrices when using only data from **D)** H3K27ac and H3K4me1, **E)** H3K27ac and H3K4me3, and **F)** H3K4me1 and H3K4me3 to annotate global patterns with the model learned using all pairs of marks. Each box corresponds to the global patterns enriched for a chromatin state annotation. The boxes use the same color scheme as the color bars in a-c and Figure 2a.

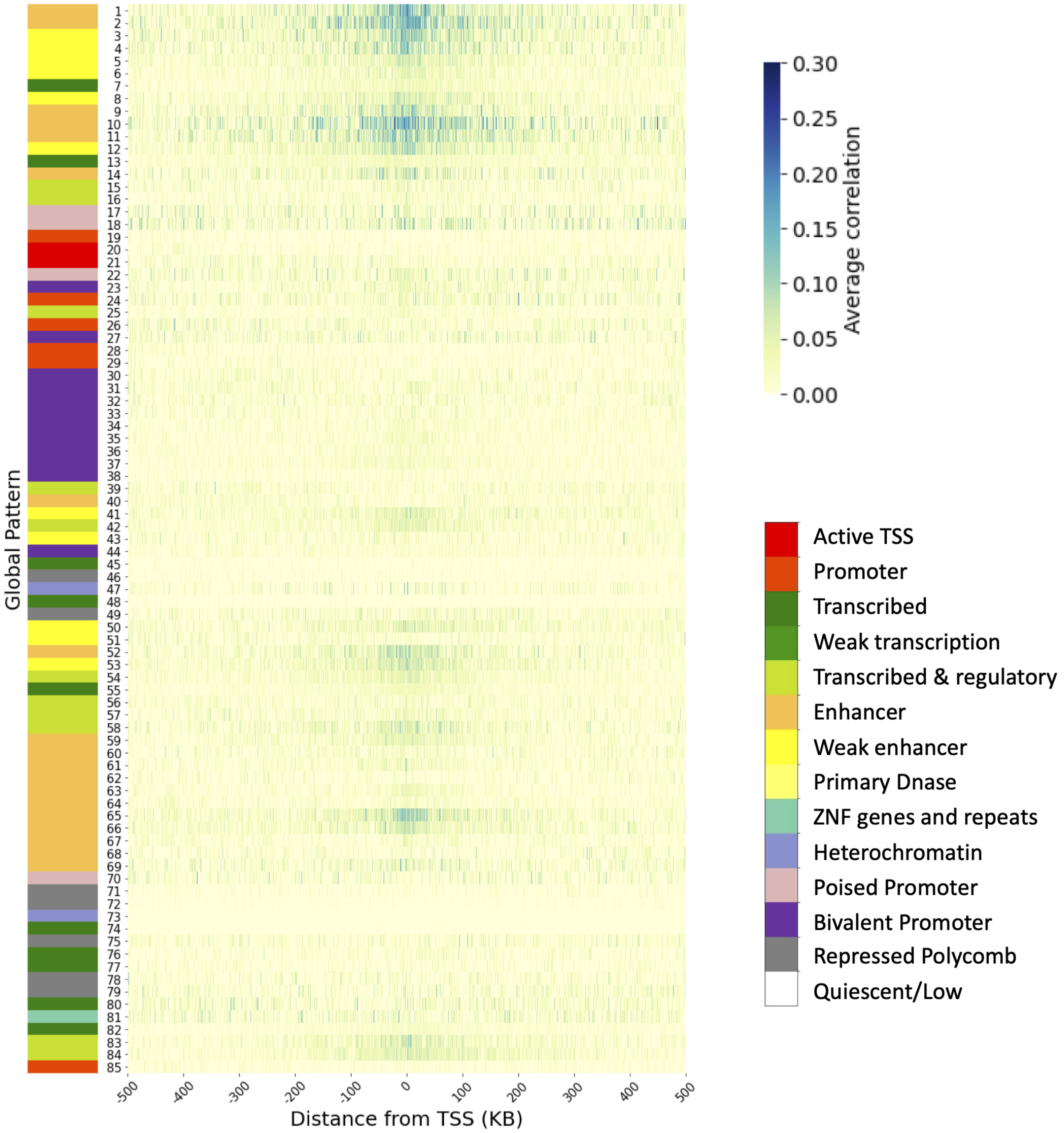

Supplementary Fig. 8: **Average correlation of H3K27ac LCL emission parameters and gene expression as a function of distance.** The genome annotation based on the LCL global patterns was used to identify genes with transcription start sites (TSS) within 500kb for each global pattern. These genes were associated with the global pattern. The average Pearson correlations between emission parameters and expression of genes across individuals are shown for each global pattern (y-axis) using only genes with TSS within each 100bp bin (x-axis). The LCL reference chromatin state[1] with the highest enrichment for each global pattern is indicated based on the color on its left and the color legend on right.

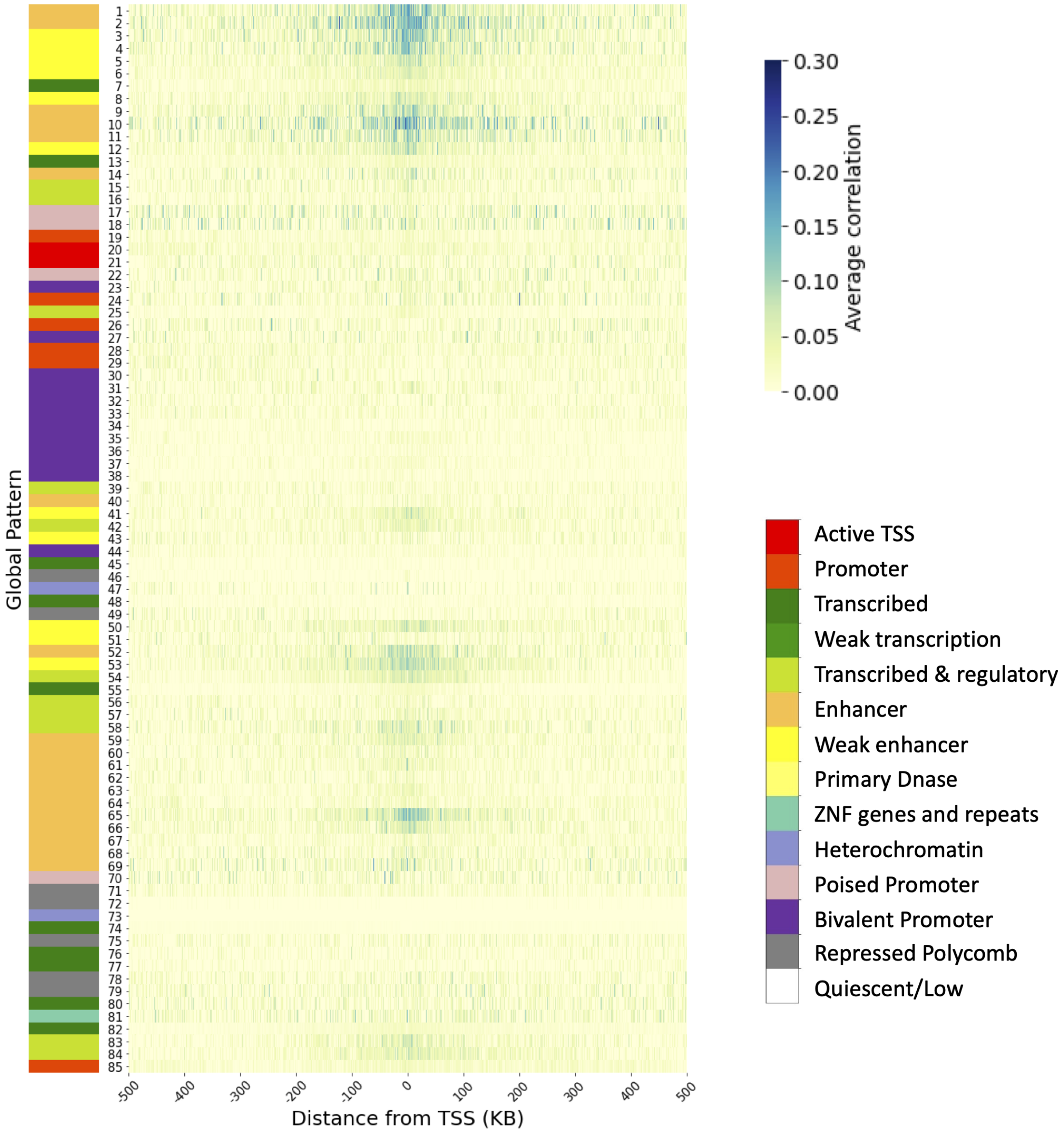

Supplementary Fig. 9: **Average correlation of H3K4me1 LCL emission parameters and gene expression as a function of distance.** The genome annotation based on the LCL global patterns was used to identify genes with transcription start sites (TSS) within 500kb for each global pattern. These genes were associated with the global pattern. The average Pearson correlations between emission parameters and expression of genes across individuals are shown for each global pattern (y-axis) using only genes with TSS within each 100bp bin (x-axis). The LCL reference chromatin state[1] with the highest enrichment for each global pattern is indicated based on the color on its left and the color legend on right.

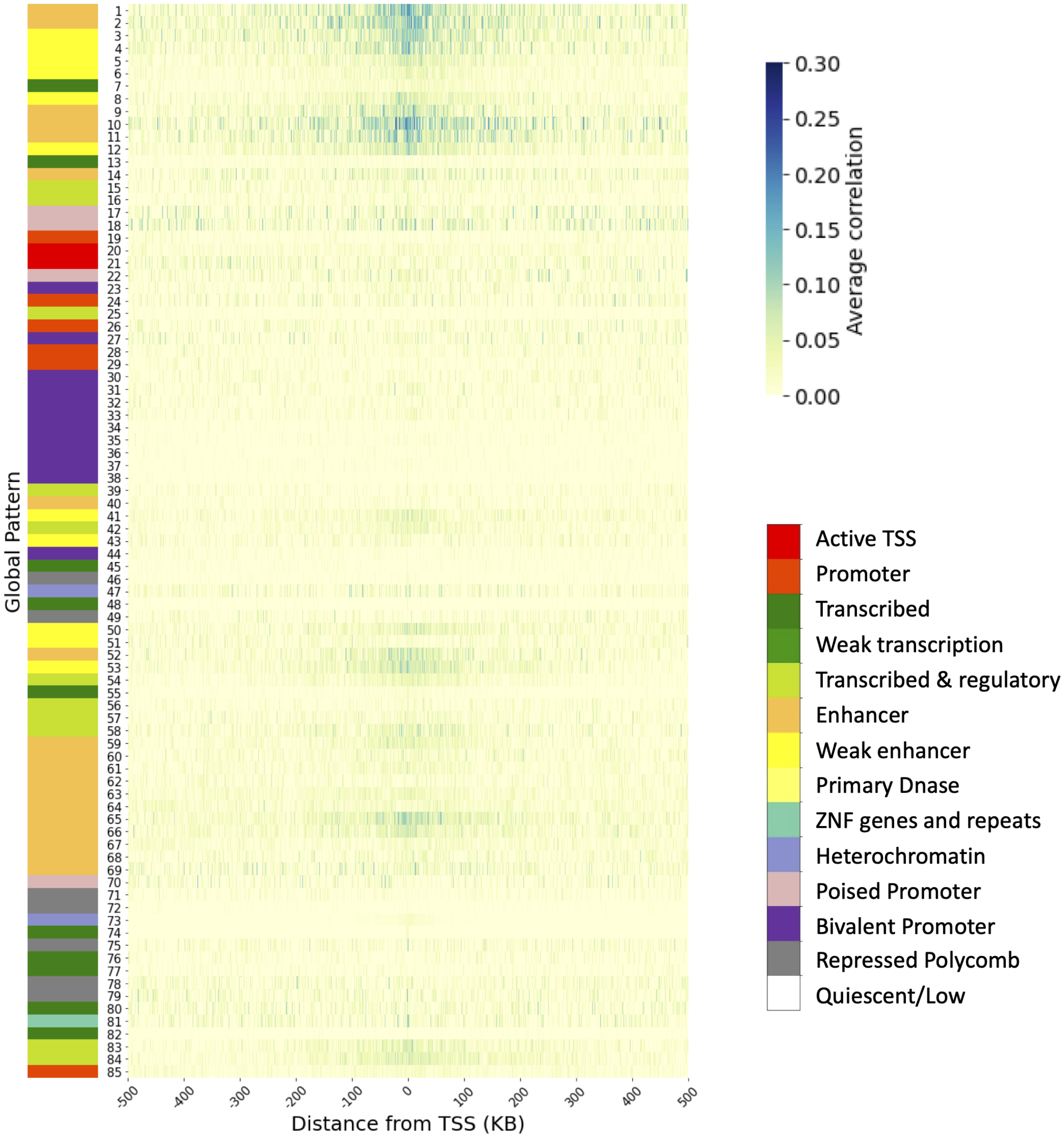

Supplementary Fig. 10: **Average correlation of H3K4me3 LCL emission parameters and gene expression as a function of distance.** The genome annotation based on the LCL global patterns was used to identify genes with transcription start sites (TSS) within 500kb for each global pattern. These genes were associated with the global pattern. The average Pearson correlations between emission parameters and expression of genes across individuals are shown for each global pattern (y-axis) using only genes with TSS within each 100bp bin (x-axis). The LCL reference chromatin state[1] with the highest enrichment for each global pattern is indicated based on the color on its left and the color legend on right.

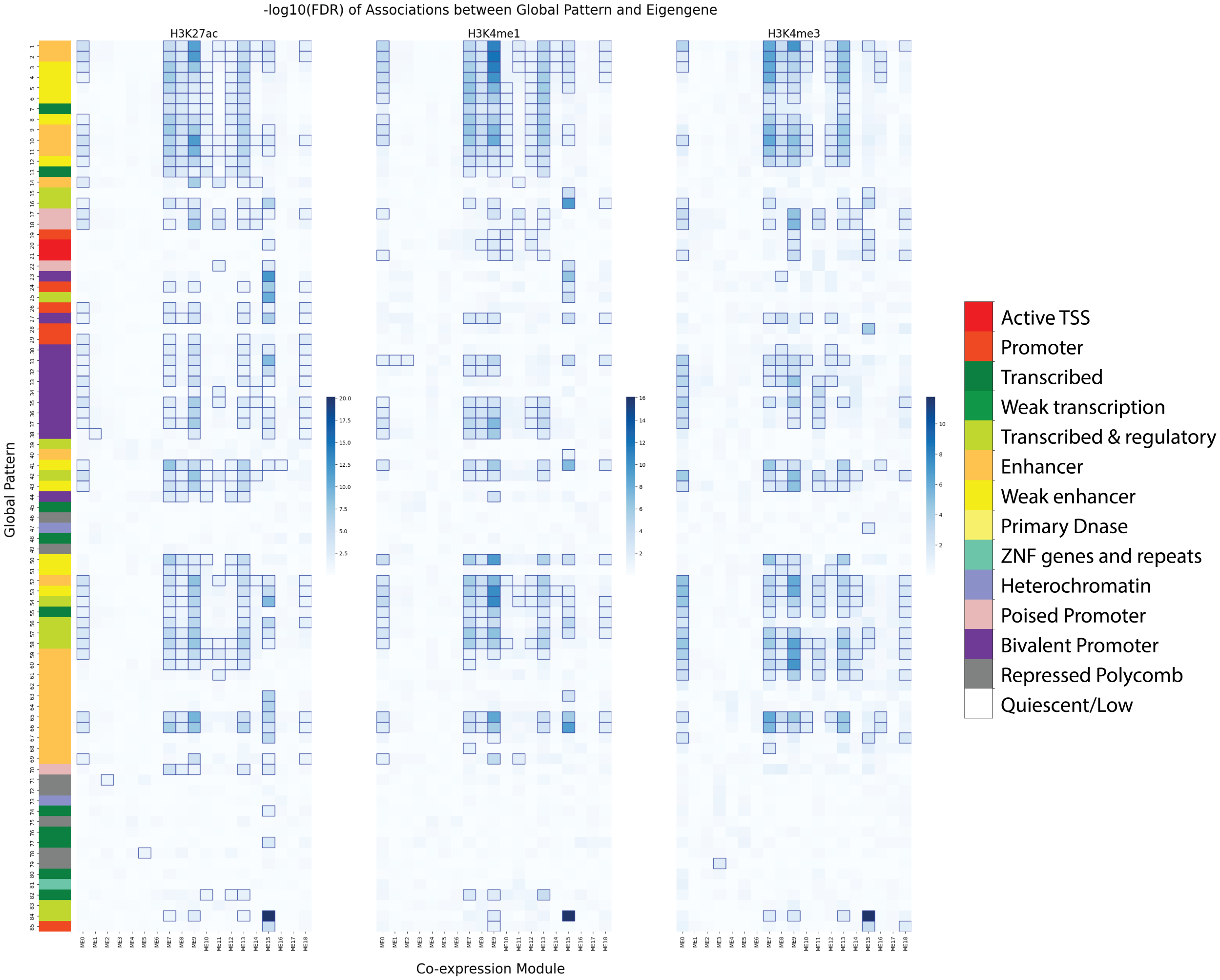

Supplementary Fig. 11: **Significance of associations between LCL emission parameters and co-expression modules.** The gene expression data was used to identify co-expression modules. The eigengene values of these modules were associated with the global patterns corresponding to each mark. The heatmaps show the -log10(FDR) of these associations for each global pattern (rows) with each co-expression module (column) for H3K27ac (left), H3K4me1 (center), and H3K27ac (right). Significant associations (FDR $< 5\%$) are outlined in blue. Each global pattern is annotated (left) with a previous LCL chromatin state annotation for one individual[1] with the highest significant enrichment (same color bar as Figure 2a).

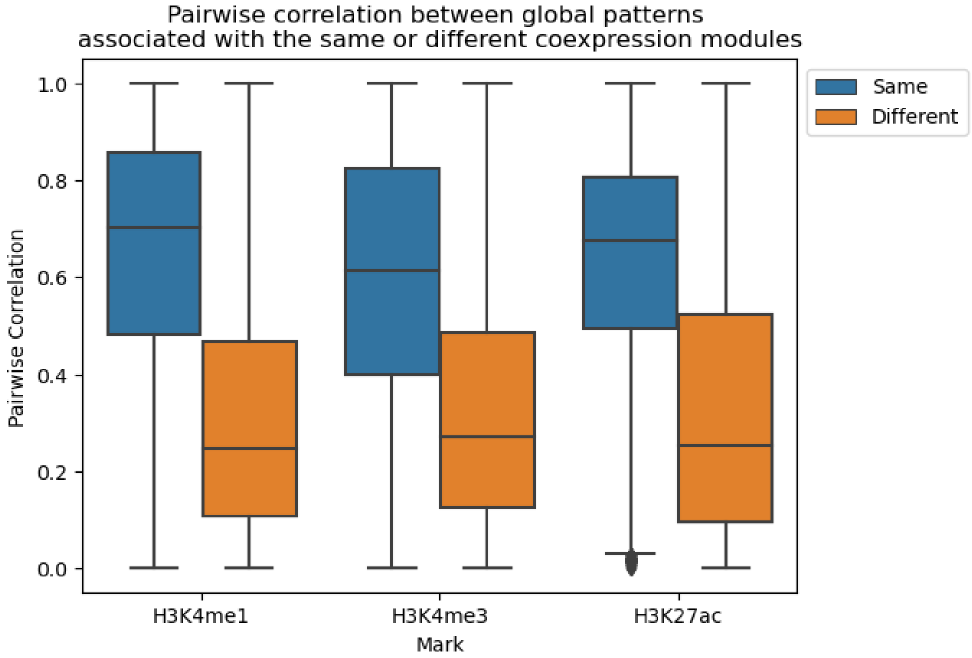

Supplementary Fig. 12: **Similarity between LCL global patterns significantly associated with the same or different co-expression modules.** Boxplots representing the pairwise correlations between emission parameters for all LCL global patterns. Emission parameters for the histone marks (H3K4me1, H3K4me3, and H3K27ac) are visualized separately. The pairwise correlations are further divided whether the pair of global patterns is significantly associated with the same or different co-expression module. 'Same' represents pairs of global patterns that are significantly associated with the same co-expression module. 'Different' represents pairs of global patterns that are significantly associated with different co-expression modules or are not significantly associated with any co-expression module.

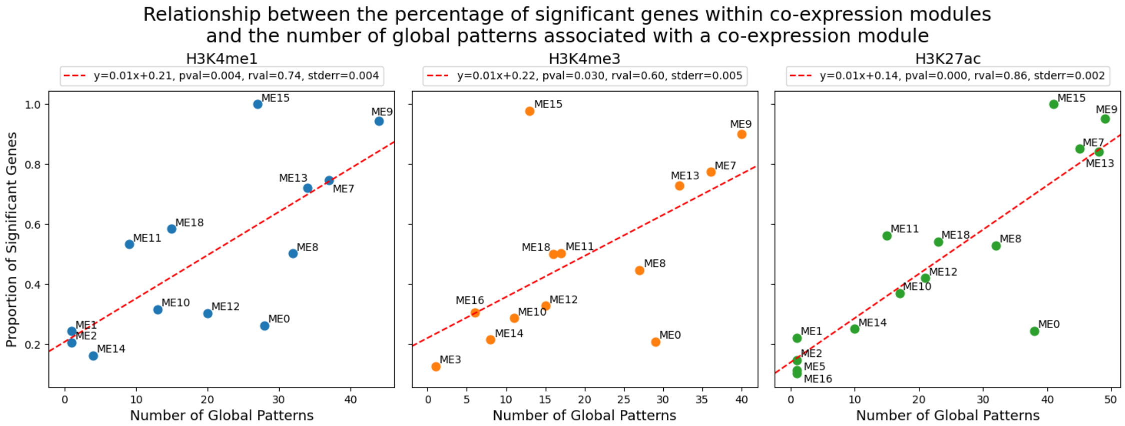

Supplementary Fig. 13: **Relationship between the percentage of significant genes within a co-expression module and the number of global patterns associated with the co-expression module**. The scatterplots show the relationship between co-expression modules and the genes assigned to each module Plots provided per mark for H3K4me1, H3K4me3, and H3K27ac (left to right). Each point represents a co-expression module. X-axis is the number of global patterns with emission parameters significantly associated with a co-expression module's eigengenes. Y-axis is the percentage of genes assigned to a co-expression module with expression significantly associated (FDR<5%) with at least one global pattern.

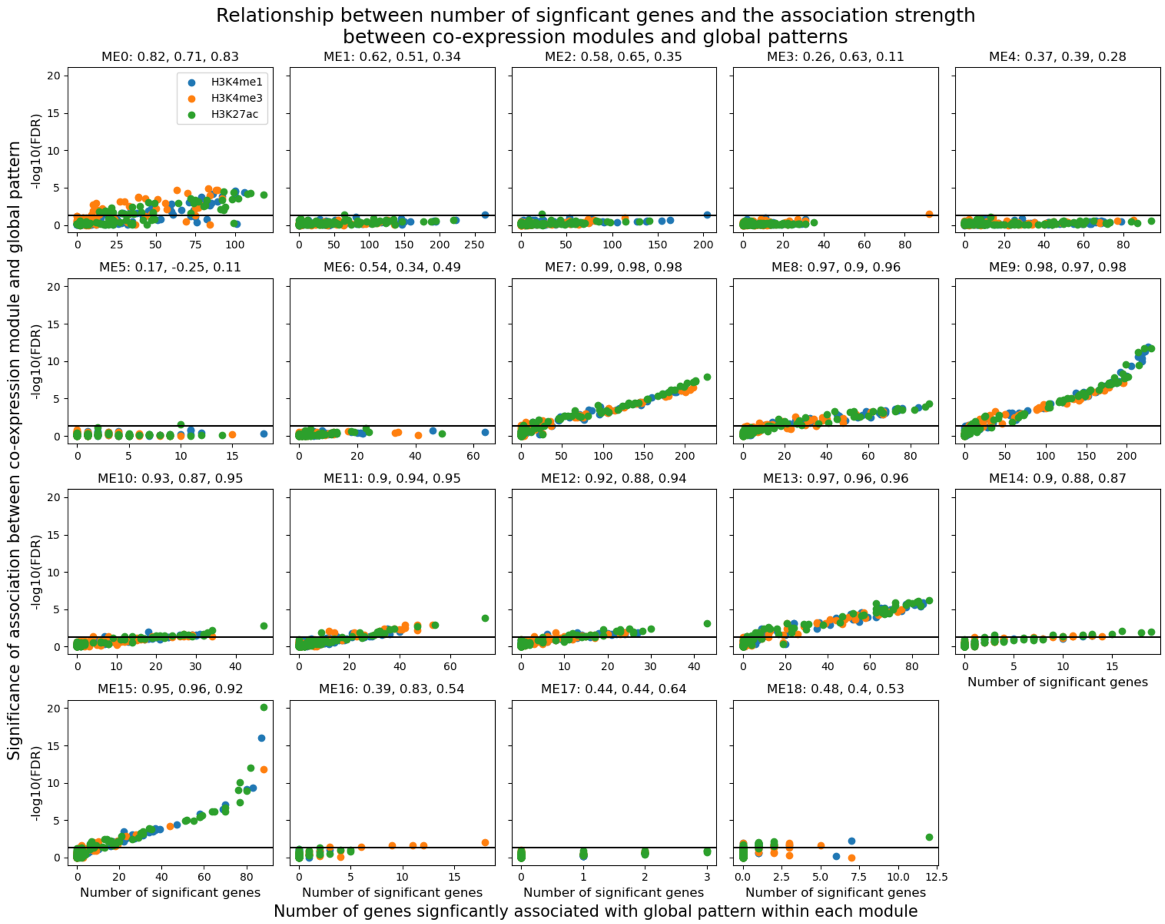

Supplementary Fig. 14: **Relationship between the number of significant genes within a co-expression module and the strength of association between a co-expression module and global pattern**. Each scatterplot shows the relationship between the number of genes significantly associated with a global pattern in a co-expression module and the strength of the association between the co-expression module and global pattern. Each point represents a global pattern. X-axis is the number of genes with expression significantly associated with a global pattern's emission parameters. Y-axis is the -log10(FDR) of the association between a co-expression module's eigengenes and a global pattern's emission parameters. Associations based on each mark (H3K4me1, H3K4me3, and H3K27ac) are colored separately within each plot. Horizontal line represents significance threshold (FDR $< 5\%$) for association test between co-expression module eigengenes and global pattern emission parameters. The title of each subplot corresponding to a single co-expression module contains the Pearson correlation between the number of significant genes and the -log10(FDR) of the module-global pattern association for H3K4me1 (left), H3K4me3 (middle), and H3K27ac (right).

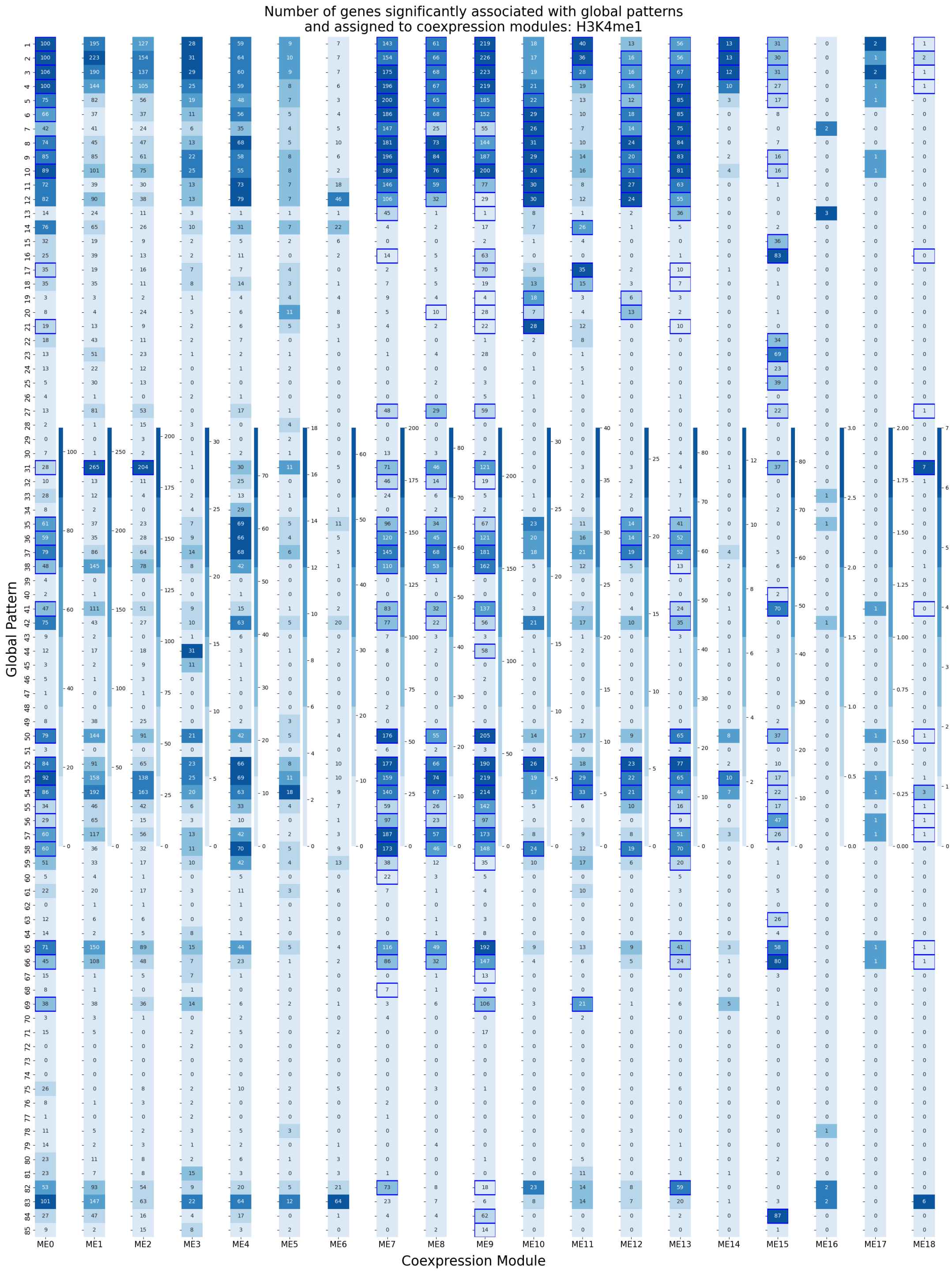

Supplementary Fig. 15: **Number of genes significantly associated with global patterns within co-expression modules for H3K4me1.** For each combination of co-expression module and global pattern, the number of genes assigned to the co-expression module with expression significantly associated with the global pattern's emission parameters for H3K4me1. Each column represents genes assigned to a co-expression module. Each row represents a global pattern. A single gene may be associated with multiple global patterns. The number in each box represents the number of genes significantly associated with a global pattern, matching the color scale from light to dark blue. Boxes are outlined if the co-expression module is significantly associated with a global pattern (same as Supplementary Fig. 11).

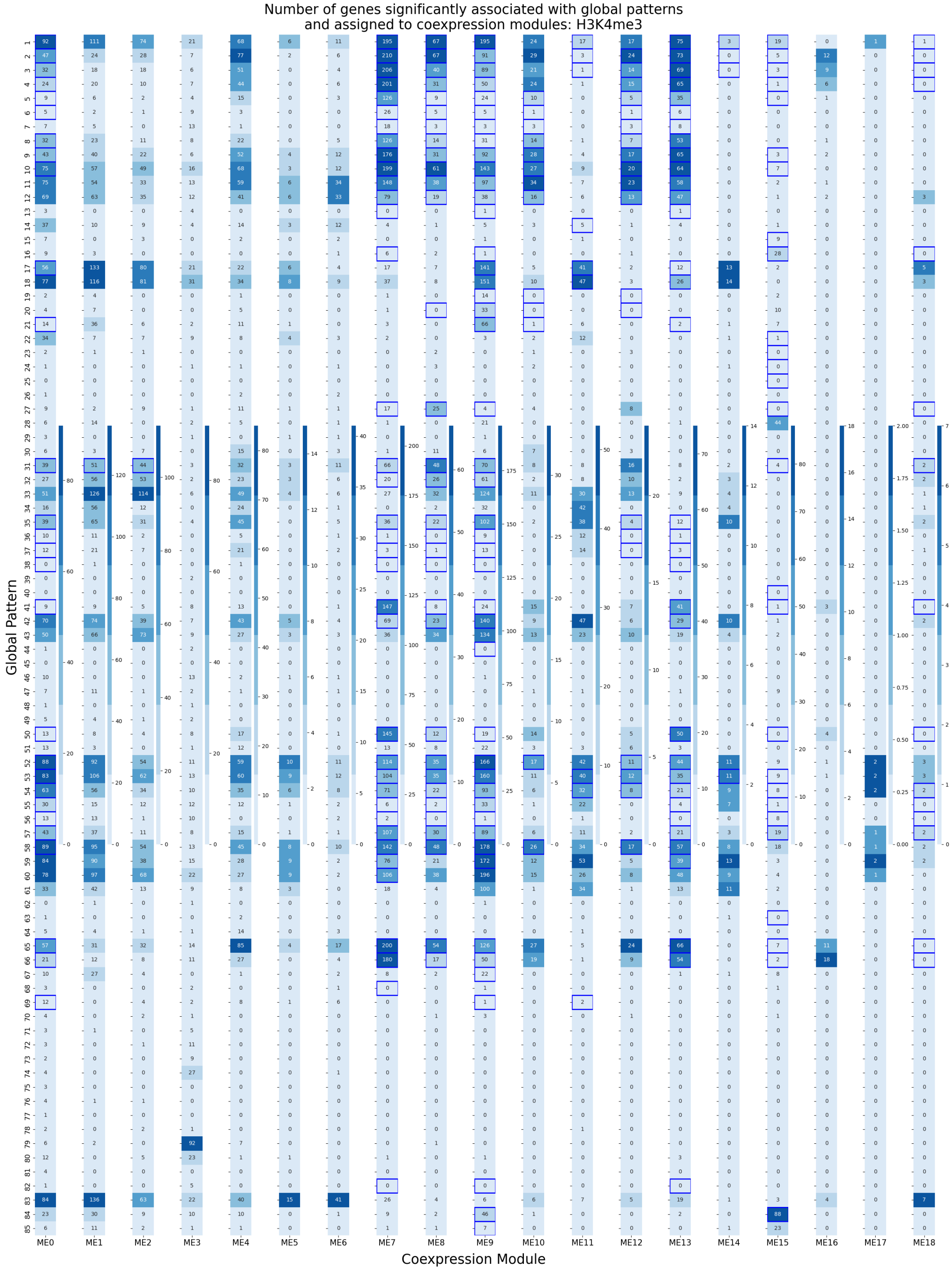

Supplementary Fig. 16: **Number of genes significantly associated with global patterns within co-expression modules for H3K4me3.** Number of genes with expression significantly associated with a global pattern's emission parameters for H3K4me3. Each column represents genes assigned to a co-expression module. A single gene may be associated with multiple global patterns. The number in each box represents the number of genes significantly associated with a global pattern, matching the color scale from light to dark blue. Boxes are outlined if the co-expression module is significantly associated with a global pattern (same as Supplementary Fig. 11).

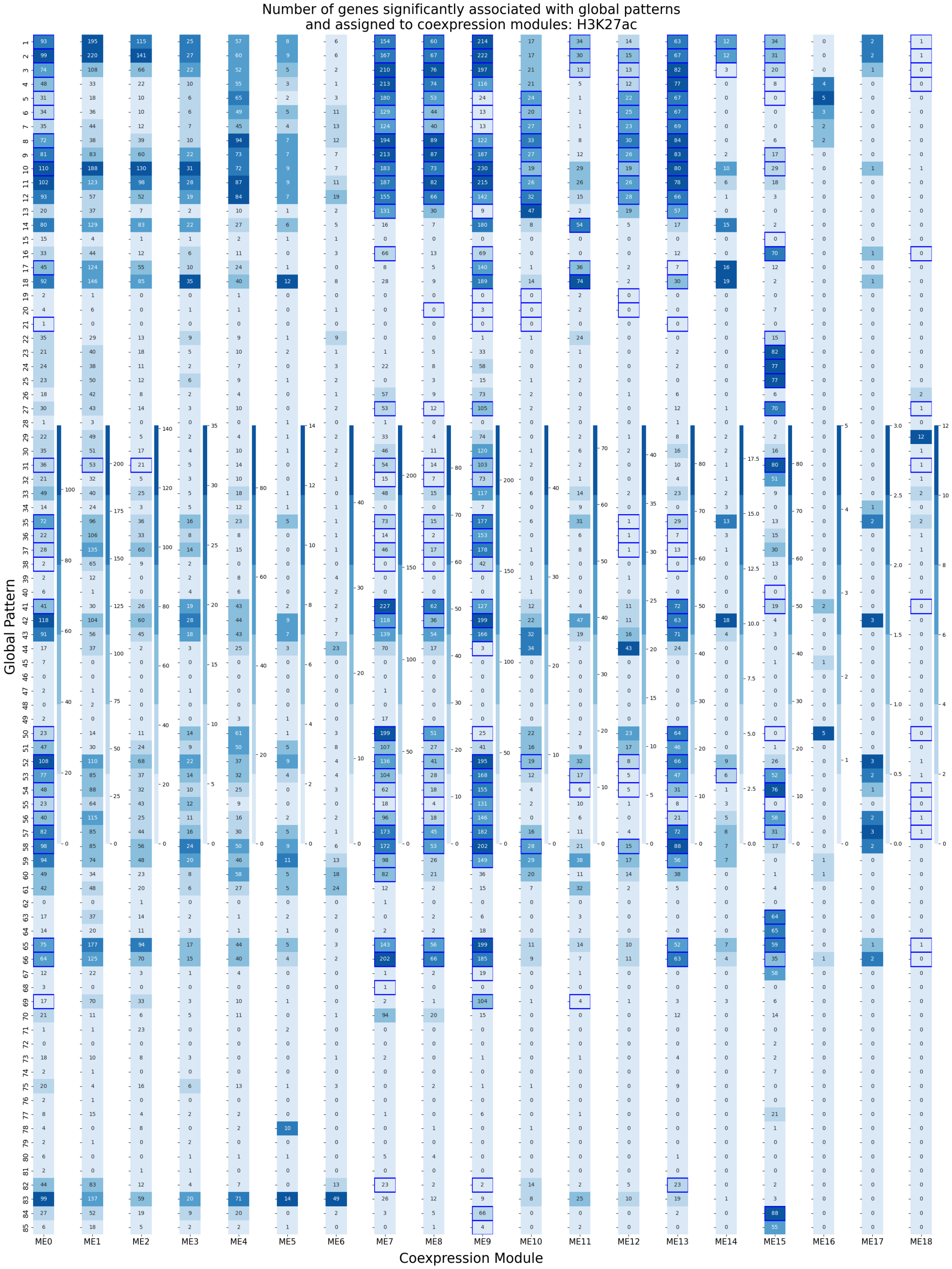

Supplementary Fig. 17: **Number of genes signficantly associated with global patterns within co-expression modules for H3K27ac.** Number of genes with expression significantly associated with a global pattern's emission parameters for H3K27ac. Each column represents genes assigned to a co-expression module. A single gene may be associated with multiple global patterns. The number in each box represents the number of genes significantly associated with a global pattern, matching the color scale from light to dark blue. Boxes are outlined if the co-expression module is significantly associated with a global pattern (same as Supplementary Fig. 11).

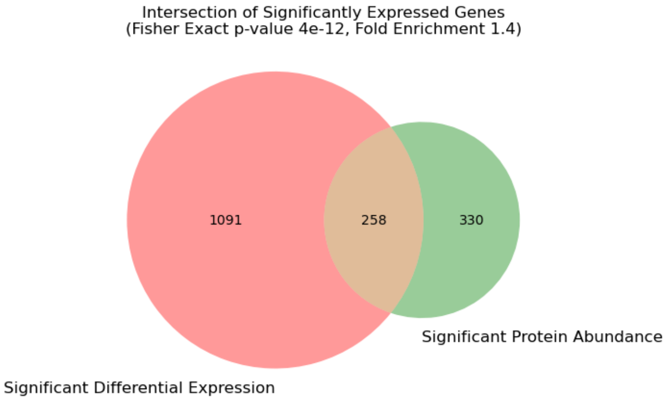

Supplementary Fig. 18: **Overlap of genes with significant differential expression and significant protein abundance for LCL data.** Venn diagram showing the number of genes with gene expression levels, protein abundance, or both significantly associated with at least one global pattern for the LCL data. This analysis only considers the 4319 genes with both protein abundance and gene expression data available with 1349 of these genes having significant differential expression and 588 having significant protein abundance. The overlap of genes between these two sets was significant (Fisher's Exact Test $p < 0.001$, Fold Enrichment 1.4).

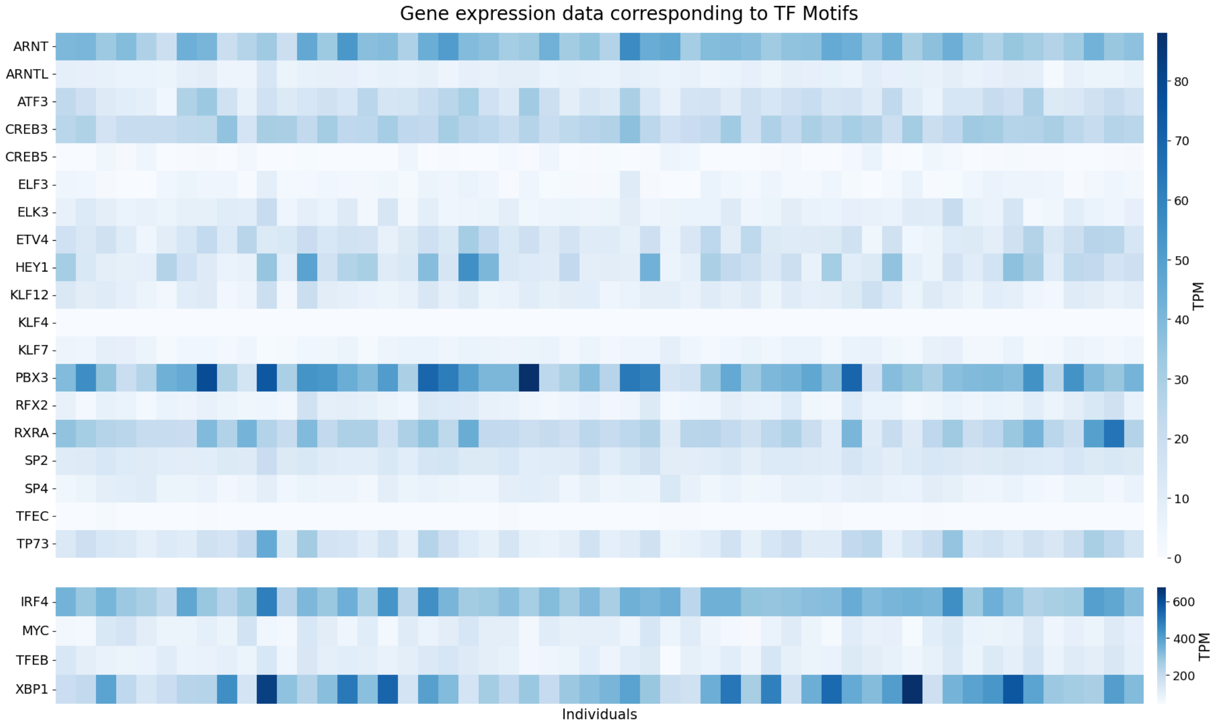

Supplementary Fig. 19: **Gene expression data for 24 TFs in LCLs across individuals**. Gene expression data for the 24 TFs shown in Figure 2c with motifs enriched in at least one global pattern (FDR<5%, -log2(fold enrichment) > 1.5) and gene expression associated with global patterns (FDR<5%). Each column represents one of the 54 individuals with available gene expression data corresponding to the order of individuals in Figure 2. Each row corresponds to one of the 24 genes. Four genes had substantially higher gene expression across individuals and are displayed in a separate heatmap with a different color scale for better visualization. Color bars represent normalized TPM levels.

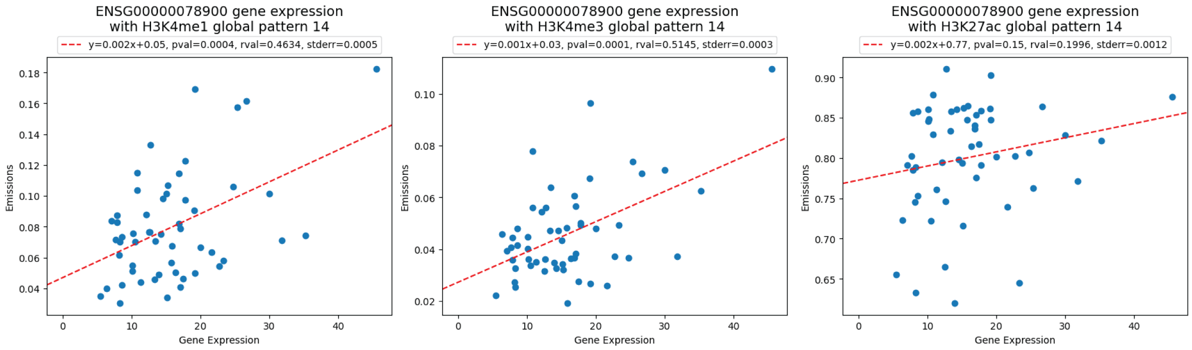

Supplementary Fig. 20: **Association between TP73 gene expression and LCL emission parameters.** Scatter plot showing the relationship between ENSG00000078900 (TP73) gene expression (x-axis) and LCL 85-state model global pattern 14 emission parameters (y-axis) for histone modifications H3K4me1 (left), H3K4me3 (center), and H3K27ac (right). Each dot corresponds to a single individual. H3K4me1 and H3K4me3 emission parameters are significantly associated with TP73 gene expression.

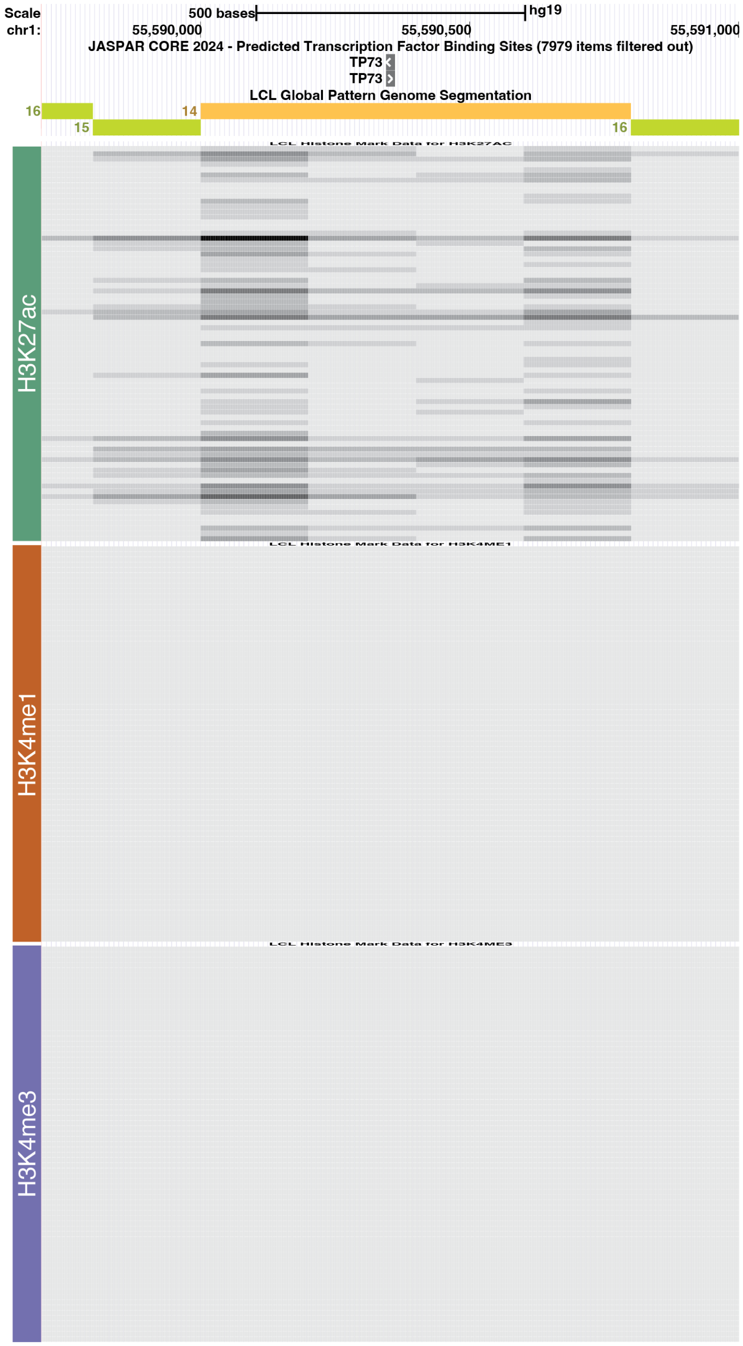

Supplementary Fig. 21: **Instance of TP73 motif in an enhancer-like global pattern.** Genome browser view showing an instance of TP73 on chromosome 1 located in a segment of the genome assigned to global pattern 14, which is associated with an enhancer chromatin state annotation. The genome browser view shows the TP73 motif present in the region above the global pattern annotations colored based on correspondence to the previous LCL chromatin state annotations in Supplementary Fig. 5. Below that is the histone modification signals across individuals for H3K27ac, H3K4me1, and then H3K4me3.

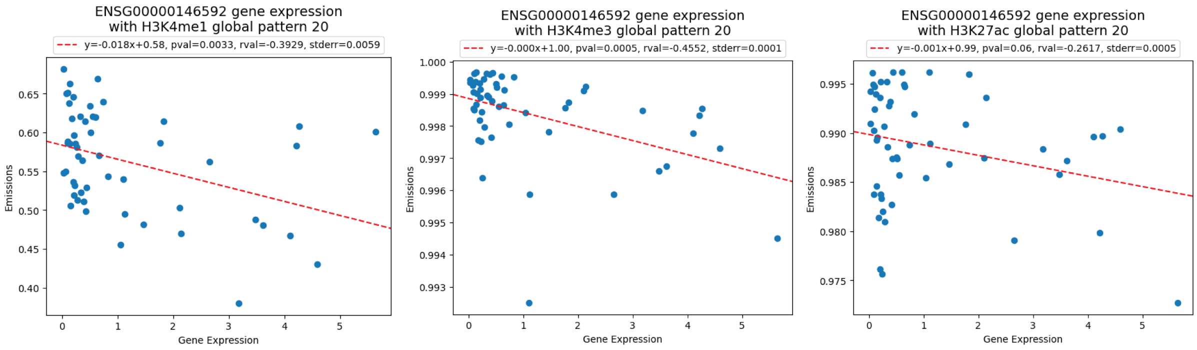

Supplementary Fig. 22: **Association between CREB5 gene expression and LCL emission parameters.** Scatter plot showing the relationship between ENSG00000146592 (CREB5) gene expression (x-axis) and LCL 85-state model global pattern 20 emission parameters (y-axis) for histone modifications H3K4me1 (left), H3K4me3 (center), and H3K27ac (right). Each dot corresponds to a single individual. H3K4me3 emission parameters are significantly associated with CREB5 gene expression.

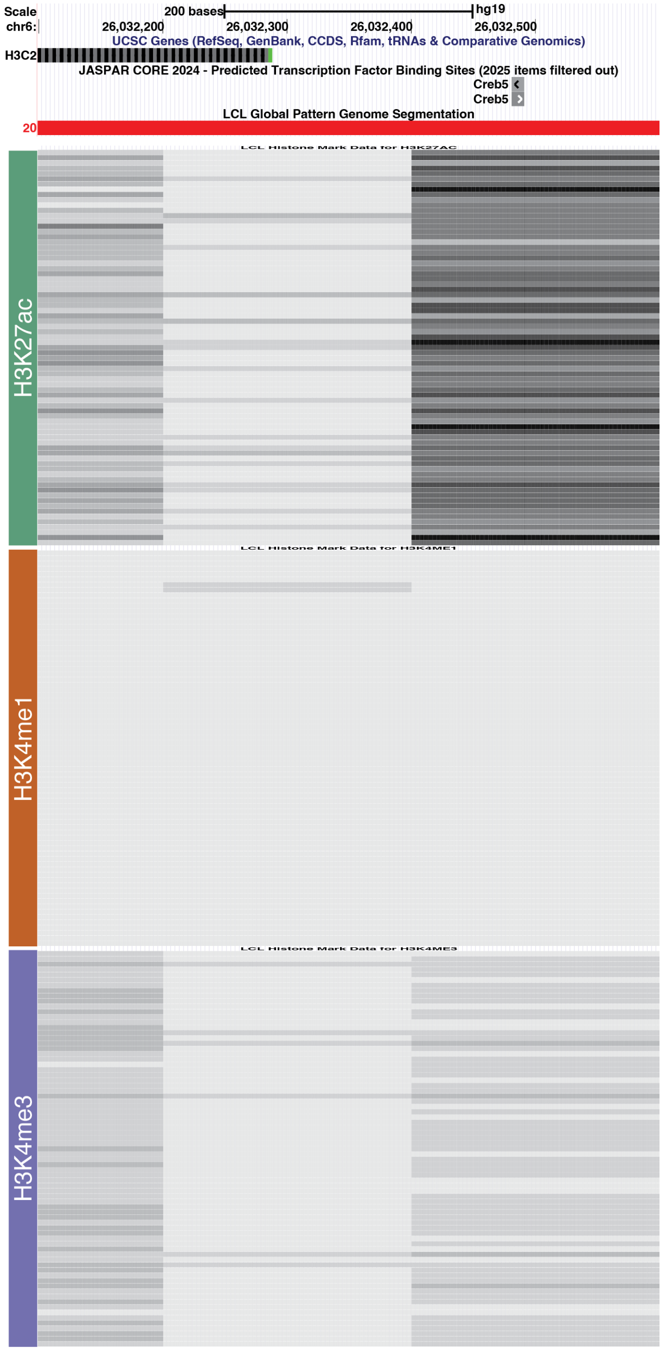

Supplementary Fig. 23: **Instance of CREB5 motif in global pattern associated with Active TSS.** Genome browser view showing an instance of CREB5 on chromosome 6 located in a segment of the genome assigned to global pattern 20, which is associated with an active TSS chromatin state annotation. The genome browser view shows genes and the CREB5 motif present in the region above the global pattern annotations colored based on correspondence to the previous LCL chromatin state annotations in Supplementary Fig. 5. Below that is the histone modification signals across individuals for H3K27ac, H3K4me1, and then H3K4me3.

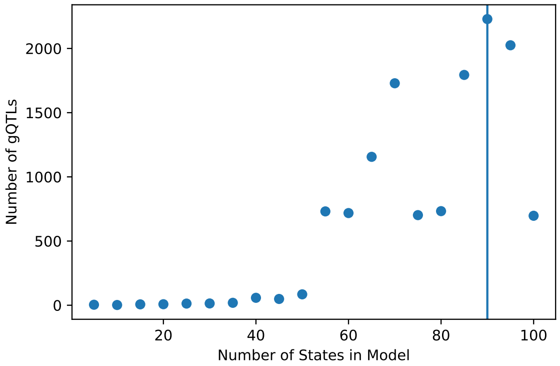

Supplementary Fig. 24: **ASD model gQTLs.** The total number of significant gQTLs (FDR<5%) is shown (y-axis) as a function of the number of states in the model (x-axis). Each point corresponds to one stacked ChromHMM model. The number of gQTLs is maximized using a 90-state model denoted with the vertical line.

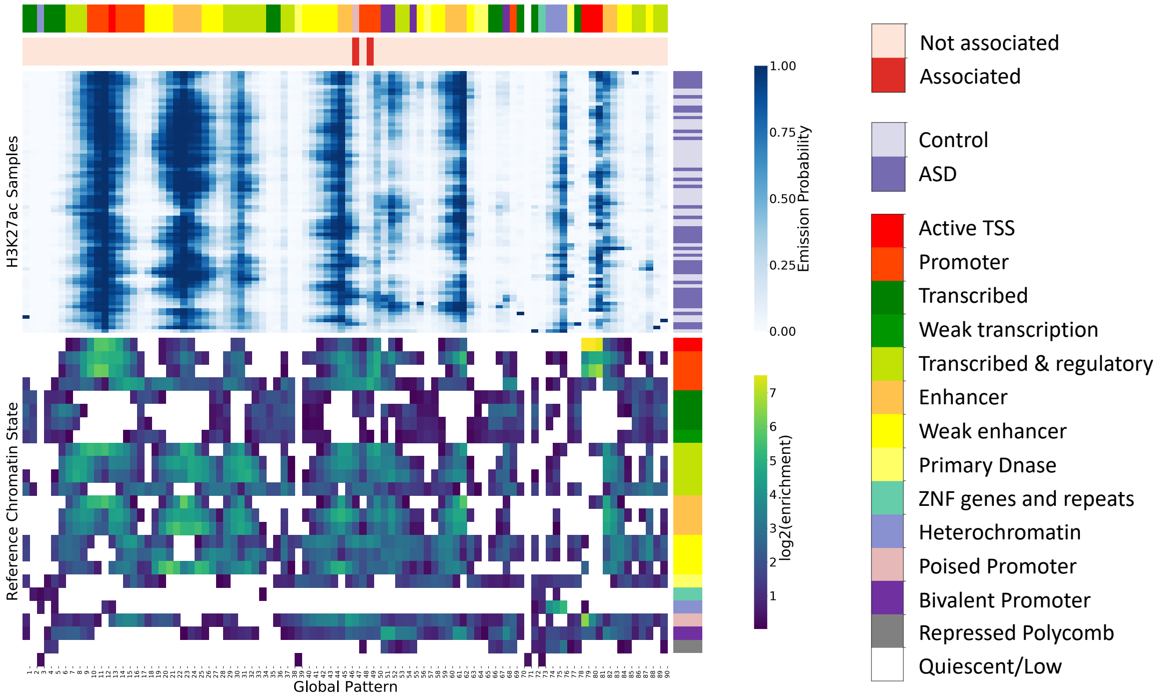

Supplementary Fig. 25: **Fold enrichment of chromatin states for ASD 90-state model.** The top heatmap shows the emission parameters learned for the 90-state model using the ASD dataset. The global patterns are on the x-axis. The samples are on the y-axis and are annotated as being either a case (dark purple) or a control (light purple) to the right. The bottom heatmap shows the log2 fold enrichment of a previous chromatin state annotation of a prefrontal cortex for a single reference epigenome using imputed data for 12-marks[1] ("Reference Chromatin State"). Significant enrichments are indicated with color (Binomial Test (two-sided), FDR<5%). The global patterns are annotated with two tracks above the emission parameters. The first shows the chromatin state from the reference prefrontal cortex chromatin state annotation with the highest enrichment. The second shows whether the state is significantly associated with ASD status (red) or not (pink). The legends for each color bar can be found on the right which includes association status (top), case/control status (middle), and chromatin state annotation (bottom).

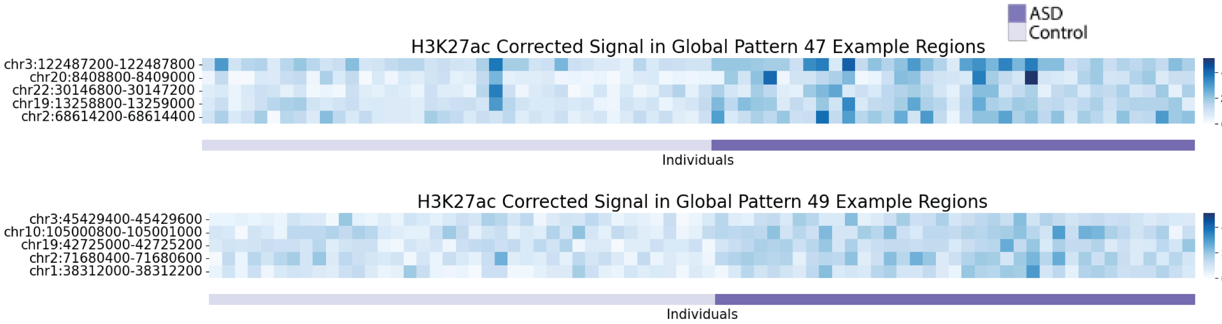

Supplementary Fig. 26: **Example windows annotated into ASD global patterns associated with ASD diagnosis status.** The figure shows example 200bp windows of the genome that were annotated into global pattern 47 (top) and 49 (bottom), both significantly associated with ASD diagnosis status. These example regions shown had the highest Area Under ROC (AUC) values between the corrected histone signal across individuals in a region and the case-control status of the individuals among 200bp windows annotated to the corresponding global pattern. Each heatmap has five rows where each row represents a 200-bp window of the genome annotated with the state of interest and each column represents an individual. The heatmap contents show the corrected histone modification signal used as input into ChromHMM, with lighter colors representing lower signal. The bar below the heatmap shows diagnosis status of each individual, with individuals sorted by diagnosis status.

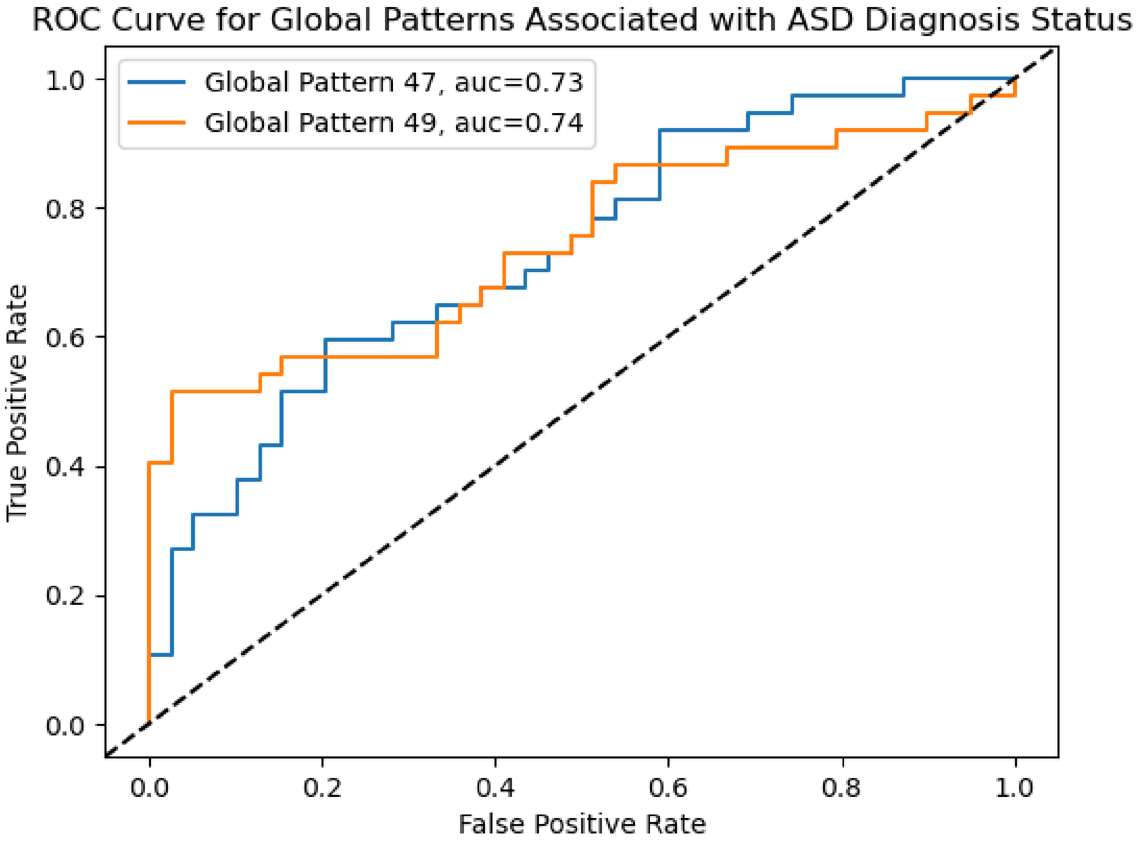

Supplementary Fig. 27: **ROC Curve for ASD global patterns associated with ASD diagnosis status.** ROC curve calculated using the emission parameters for global patterns 47 and 49, both significantly associated with ASD diagnosis status. ROC curve calculated using the emission parameters for each global pattern across all individuals. AUC score is in legend.

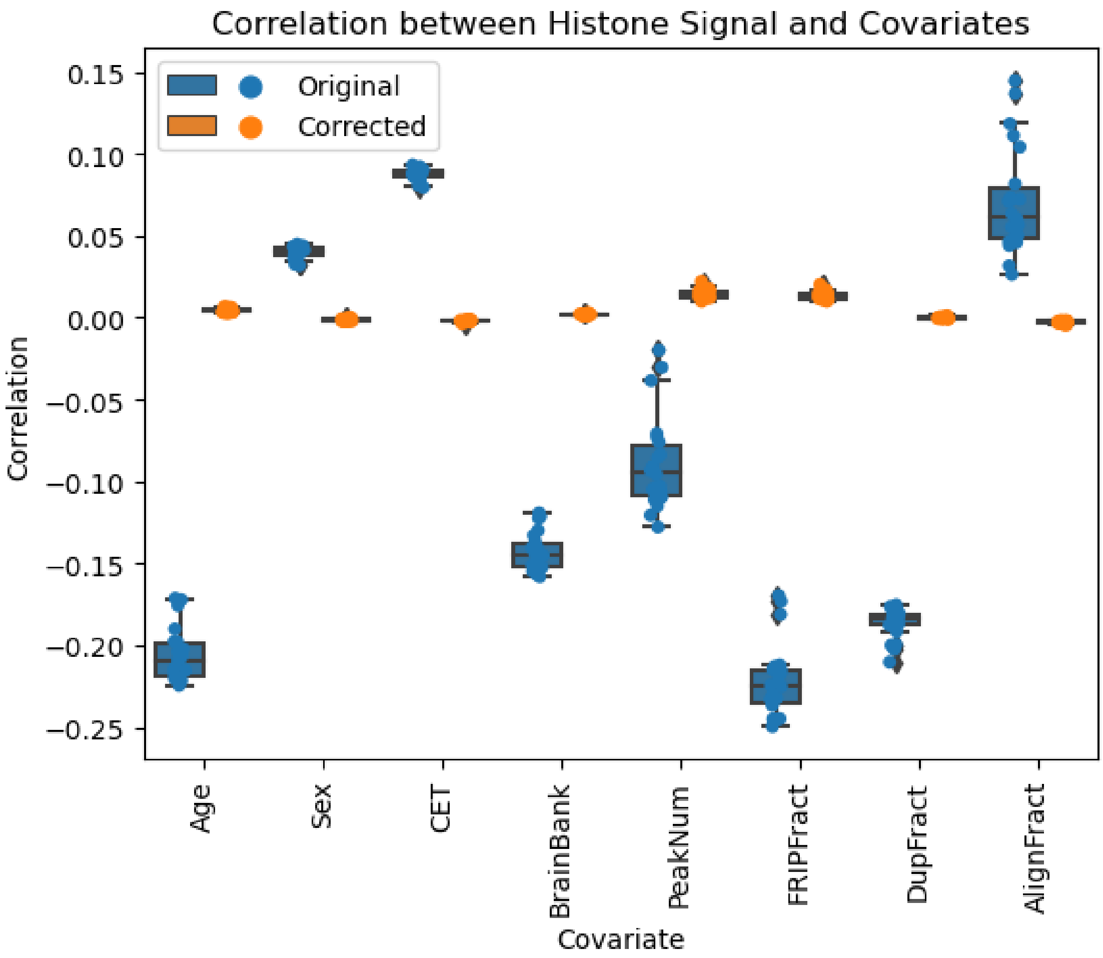

Supplementary Fig. 28: **Correlation of histone signal and covariate values in ASD dataset.** Boxplot representing the mean correlation between histone signals and covariates across all individuals (n=75) before and after covariate correction across histone data. Each dot represents the average correlation across genomic bins within a single chromosome. The covariates Age, Sex, CET (percentage of neuronal cells), Brain Bank, PeakNum (number of peaks), FRIPFract (fraction of reads in peak), DupFract (read duplicate fraction), and AlignFract (read alignment fraction) are accounted for during signal correction. The correlation between signal and covariates is decreased after signal correction.

GO term	Total	Obs	Exp	Fold Enrichment	P	FDR
cellular response to interleukin-1	114	9	1.53	5.89	3.85E-05	3.01E-02
rRNA processing	219	12	2.94	4.09	6.27E-05	4.46E-02
macromolecule modification	2922	65	39.17	1.66	4.04E-05	3.02E-02
protein metabolic process	3900	82	52.28	1.57	1.92E-05	1.68E-02
cellular macromolecule metabolic process	4497	92	60.28	1.53	1.37E-05	1.27E-02

Supplementary Table 1: **GO enrichment of genes associated with global pattern 49 in the ASD model.** We performed GO enrichment of the 316 genes associated with global pattern 49 in the ASD model using the whole genome as the background. The terms shown ("GO term") were significantly enriched after correcting for multiple testing using an FDR threshold of 5%. The total number of genes for the term ("Total"), the observed number of genes in the foreground ("Obs"), the expected number of genes ("Exp"), the fold enrichment ("Fold Enrichment"), the raw p-value ("P"), and the FDR corrected p-value ("FDR") are shown in the table above.

Supplementary Reference

1. Ernst, J. & Kellis, M. Large-scale imputation of epigenomic datasets for systematic annotation of diverse human tissues. *Nature Biotechnology* **33,** 364–376 (2015).
