# Supplementary material for: Genome-wide identification and analysis of recurring patterns of epigenetic variation across individuals
Source: Commun Biol. 2025 Jun 7;8:888. doi: 10.1038/s42003-025-08179-5 (PMC12145423; doi:10.1038/s42003-025-08179-5)
Supplement: Supplementary file 7 — Description of additional supplementary files [file 42003_2025_8179_MOESM7_ESM.docx]

Description of Additional Supplementary Files

**File name:** Supplementary Data 1

**Description:** List of samples, model parameters, and model validation.

**File name:** Supplementary Data 2

**Description:** List of LCL and ASD gQTLs, GO enrichments, and LCL eQTLs.

**File name:** Supplementary Data 3

**Description:** LCL and ASD global pattern genome annotation.

**File name:** Supplementary Data 4

**Description:** Significantly enriched TF motifs, external chromatin states, external annotations, and potential trans-regulators in LCL and ASD datasets.

**File name:** Supplementary Data 5

**Description:** Significant associations with large-scale molecular variation including gene expression data, co-expression modules, and protein quantification data.
