# Supplementary material for: Genome-wide identification and analysis of recurring patterns of epigenetic variation across individuals
Source: Commun Biol. 2025 Jun 7;8:888. doi: 10.1038/s42003-025-08179-5 (PMC12145423; doi:10.1038/s42003-025-08179-5)
Supplement: Supplementary file 8 — Reporting summary [file 42003_2025_8179_MOESM8_ESM.pdf]

nature portfolio

Corresponding author(s): Jason Ernst

Last updated by author(s): Apr 30, 2025

Reporting Summary

Nature Portfolio wishes to improve the reproducibility of the work that we publish. This form provides structure for consistency and transparency in reporting. For further information on Nature Portfolio policies, see our Editorial Policies and the Editorial Policy Checklist.

Statistics

For all statistical analyses, confirm that the following items are present in the figure legend, table legend, main text, or Methods section.

n/a	Confirmed	
☐	☒	The exact sample size (*n*) for each experimental group/condition, given as a discrete number and unit of measurement
☒	☐	A statement on whether measurements were taken from distinct samples or whether the same sample was measured repeatedly
☐	☒	The statistical test(s) used AND whether they are one- or two-sided<br>*Only common tests should be described solely by name; describe more complex techniques in the Methods section.*
☐	☒	A description of all covariates tested
☐	☒	A description of any assumptions or corrections, such as tests of normality and adjustment for multiple comparisons
☐	☒	A full description of the statistical parameters including central tendency (e.g. means) or other basic estimates (e.g. regression coefficient) AND variation (e.g. standard deviation) or associated estimates of uncertainty (e.g. confidence intervals)
☐	☒	For null hypothesis testing, the test statistic (e.g. *F*, *t*, *r*) with confidence intervals, effect sizes, degrees of freedom and *P* value noted<br>*Give P values as exact values whenever suitable.*
☒	☐	For Bayesian analysis, information on the choice of priors and Markov chain Monte Carlo settings
☒	☐	For hierarchical and complex designs, identification of the appropriate level for tests and full reporting of outcomes
☐	☒	Estimates of effect sizes (e.g. Cohen's *d*, Pearson's *r*), indicating how they were calculated

Our web collection on statistics for biologists contains articles on many of the points above.

Software and code

Policy information about availability of computer code

Data collection	All data used was previously published.
Data analysis	Data preprocessing code can be found at https://github.com/jzou1115/EpiVarIndividuals. Chromatin state learning was performed using ChromHMM (v1.11), which is available at https://ernstlab.github.io/ChromHMM/. We performed the quantitative trait loci analysis using the MatrixQTL software (v2.2).

For manuscripts utilizing custom algorithms or software that are central to the research but not yet described in published literature, software must be made available to editors and reviewers. We strongly encourage code deposition in a community repository (e.g. GitHub). See the Nature Portfolio guidelines for submitting code & software for further information.

Data

Policy information about availability of data

All manuscripts must include a data availability statement. This statement should provide the following information, where applicable:

- Accession codes, unique identifiers, or web links for publicly available datasets
- A description of any restrictions on data availability
- For clinical datasets or third party data, please ensure that the statement adheres to our policy

Our work integrates data from a number of published data, including LCL ChIP-seq and RNA-seq data (http://mitra.stanford.edu/kundaje/portal/chromovar3d/, a link to a copy of the data we used is available through https://github.com/ernstlab/EpiVarIndividuals/), LCL protein data, ASD ChIP-seq data (https://

www.synapse.org/#!Synapse:syn4587616), and ASD RNA-seq data (https://www.synapse.org/#!Synapse:syn11242290).

A list of samples in each of the LCL datasets, model parameters learned in the paper from the LCL, ASD, and BLUEPRINT data, and model validation results can be found in Supplementary Data 1. The significantly enriched gQTLs in the LCL and ASD data, GREAT enrichments for the gQTLs, significantly enriched eQTLs that overlap with gQTLs in the LCL dataset, and BLUEPRINT gQTL replication statistics can be found in Supplementary Data 2. The global pattern annotation of the LCL and ASD models can be found in Supplementary Data 3. The LCL and ASD TF motif enrichments, enrichments with external chromatin states and annotations, and potential trans-regulators can be found in Supplementary Data 4. The significant associations between the LCL and ASD global patterns and gene expression data, co-expression module information and significant associations with global patterns, the significant associations between LCL global patterns and protein quantification data, the overlap between significantly associated genes and proteins, the correlation between gene expression and global patterns, and the association between ASD emission parameters and diagnosis status can be found in Supplementary Data 5.

Research involving human participants, their data, or biological material

Policy information about studies with human participants or human data. See also policy information about sex, gender (identity/presentation), and sexual orientation and race, ethnicity and racism.

Reporting on sex and gender	Sex meta data for samples was collected and published in prior studies.
Reporting on race, ethnicity, or other socially relevant groupings	The lymphoblastoid cell line (LCL) data was collected in Yoruban samples. While the autism spectrum disorder (ASD) data contains Caucasian, Asian, African American, and mixed ancestry samples.
Population characteristics	The lymphoblastoid cell line (LCL) data was collected in Yoruban samples. While the autism spectrum disorder (ASD) data contains Caucasian, Asian, African American, and mixed ancestry samples.
Recruitment	N/A - We used previously published data for this work.
Ethics oversight	N/A - We used previously published data for this work.

Note that full information on the approval of the study protocol must also be provided in the manuscript.

Field-specific reporting

Please select the one below that is the best fit for your research. If you are not sure, read the appropriate sections before making your selection.

☒ Life sciences ☐ Behavioural & social sciences ☐ Ecological, evolutionary & environmental sciences

For a reference copy of the document with all sections, see nature.com/documents/nr-reporting-summary-flat.pdf

Life sciences study design

All studies must disclose on these points even when the disclosure is negative.

Sample size	Our chromatin state models use histone modification data in lymphoblastoid cell line (75 samples with 3 histone modifications per sample) and ASD cases and controls (76 samples with 1 histone modification per sample)
Data exclusions	Only samples excluded from previously published works were excluded from analysis.
Replication	We identified gQTLs in the LCL data set. We analyzed the replication of our gQTL findings in an independent data set from BLUEPRINT.
Randomization	N/A - We used previously published data for this work.
Blinding	N/A - We did not perform a clinical study.

Reporting for specific materials, systems and methods

We require information from authors about some types of materials, experimental systems and methods used in many studies. Here, indicate whether each material, system or method listed is relevant to your study. If you are not sure if a list item applies to your research, read the appropriate section before selecting a response.

Materials & experimental systems

n/a	Involved in the study
☒	☐ Antibodies
☒	☐ Eukaryotic cell lines
☒	☐ Palaeontology and archaeology
☒	☐ Animals and other organisms
☒	☐ Clinical data
☒	☐ Dual use research of concern
☒	☐ Plants

Methods

n/a	Involved in the study
☒	☐ ChIP-seq
☒	☐ Flow cytometry
☒	☐ MRI-based neuroimaging

Plants

Seed stocks	N/A
Novel plant genotypes	N/A
Authentication	N/A
